# Obesity, physical activity and prediabetes in adult children of people with diabetes ^1^

**DOI:** 10.1590/1518-8345.2102.2981

**Published:** 2018-01-08

**Authors:** Lidia G. Compeán-Ortiz, Laura Elena Trujillo-Olivera, Ana María Valles-Medina, Eunice Reséndiz-González, Beatriz García-Solano, Beatriz Del Angel Pérez

**Affiliations:** 2 PhD, Researcher Professor, Facultad de Enfermería Tampico, Universidad Autónoma de Tamaulipas, Tampico, Tamaulipas, Mexico.; 3 PhD, Researcher Professor, Centro Mesoamericano de Estudios en Salud Pública y Desastres, Universidad Autónoma de Chiapas, Tuxtla Gutiérrez, Chiapas, Mexico.; 4 PhD, Researcher Professor, Facultad de Medicina y Psicología, Universidad Autónoma de Baja California, Tijuana, Baja California, Mexico.; 5 PhD, Researcher Professor, Facultad de Enfermería, Benemérita Universidad Autónoma de Puebla, Puebla, Mexico.; 6 MSc, Researcher Professor, Facultad de Enfermería Tampico, Universidad Autónoma de Tamaulipas, Tampico, Tamaulipas, Mexico.

**Keywords:** Obesity, Physical Activity, Prediabetes, Family, Diabetes, Nursing

## Abstract

**Objectives:**

Determine prevalence of obesity / overweight, physical activity (PA) and prediabetes in adult children of parents with type 2 diabetes; identify differences according to sociodemographic variables, and describe the relationship of obesity/overweight with fasting glucose (FG) and glycosylated hemoglobin (A1C).

**Methods:**

Cross-sectional study in 30 Mexican families with 53 participating adult children. Obesity / overweight was determined with Body Mass Index (BMI), Waist Circumference (WC) and body fat percentage (BFP); PA with the short International Physical Activity Questionnaire (IPAQ), and prediabetes with FG.

**Results:**

64% of participants presented obesity / overweight, 32% low PA, and 19% prediabetes. Men had higher WC than women (U= 219, p= 0.03). Women showed more BFP than men (U= 142, p <0.01). Blood glucose was related to BFP (rs= 0.336, p < 0.05), the A1C with the BMI (rs= 0.417, p <0.01), WC (rs= 0.394, p<0.01), BFP (rs= 0.494, p<0.01) and intense PA (rs= - 0.285, p<0.05).

**Conclusions:**

High prevalence of obesity / overweight and low PA were found. The FG was related only to BFP and A1C, in addition to BMI, WC and inversely with intense BP. It is recommended to modify the educational strategies of nursing at a family level.

## Introduction

Diabetes mellitus type 2 (DM2) is a progressive public health problem parallel to population aging, increased urbanization, and changes in lifestyles. It is estimated that there are approximately 415 million people in the world with this disease; it is projected that this amount could increase to 642 million by 2040^1^. 

The prevalence of diabetes in Latin America fluctuates between 8 - 10% in people over 20 years of age^2^. In Mexico, the National Health and Nutrition Survey of 2012 reported a prevalence of 9.2%, equivalent to 6.4 million people. The states with the highest prevalence (from 10. 2 - 12.3%) were the Federal District, Nuevo León, Veracruz, Estado de México, Tamaulipas, Durango, and San Luis Potosí^3^.

Before a person manifests DM2, a condition known as prediabetes is present and is characterized by blood glucose levels that are higher than normal, but not high enough to diagnose diabetes. According to the American Diabetes Association^4^, the diagnosis of prediabetes is established through plasma glucose: 1) with the fasting glucose test between 100 - 125 mg / dl, or the glucose tolerance test two hours after an oral load of 75 g of anhydrous glucose in 300 ml of water, ingested in less than five minutes, with a value from 140 - 199 mg/dl, and 2) through glycosylated hemoglobin (A1c) with a value of 5.7 to 6.4% as long as a method certified by the National Standardization Program of Glycohemoglobin is used.

Prediabetes is a health problem in diverse populations; in the United States, a national prevalence of 35.5% was reported in adults (> 20 years of age) between 1999-2010^5^; in England, a prevalence of 35.5%^6^; in China, of 15.5%^7^; in Spain, of 14.8%^8^; and in Bangladesh, a prevalence of 22.4%^9^. In Mexico, the National Survey of 2006 reported an altered fasting glucose of 20.1%, representing 16 million Mexicans at risk of advancing to the diabetic state^2^. Likewise, some authors have reported a prevalence of prediabetes in urban and rural Mexican population of 19.9%^10^. 

Most people with impaired glucose will develop overt DM2 over a ten-year period if something is not done to prevent it^2^. Because the evolution of prediabetes is delayed mainly using interventions for modifiable risk factors, avoiding up to 58% progression to diabetes^11^, it is necessary to evaluate risks and design interventions directed at the fundamental variables for the control of DM2: obesity / overweight, physical inactivity and inadequate nutrition^12^.

The most used parameter to assess obesity is the Body Mass Index (BMI), as well as waist circumference, which can better predict the risks of cardiovascular comorbidity. Abdominal adiposity is related to metabolic and cardiovascular alterations secondary to obesity; adipose tissue during obesity has been associated with insulin resistance and with diabetes mellitus^13^. Electrical impedance is a method to evaluate body composition that estimates total body fat, which can also influence health risks.

Physical inactivity is the fourth risk factor for global mortality, and is estimated to be the main cause of 27% of diabetes cases^14^. Physical activity helps control body weight, and decreases the risk of type 2 diabetes, the risk of cardiovascular diseases, and metabolic syndrome. According to its intensity, PA can be mild, moderate or intense; the higher the performance, the greater the benefits. On the other hand, it is convenient to point out that regardless of the PA performed, spending a lot of time on sedentary behaviors (which are not presented in this work) can additionally increase health risks.

Inadequate nutrition also requires special attention. In each of the stages of the life cycle, it is necessary to maintain a balance between the energy consumed and the energy expended. The improvement of dietary habits is a problem for the whole society, and not only each of the individuals that compose it; therefore, it requires a population-based, multisectoral, multidisciplinary approach adapted to cultural circumstances^15^. It is recognized that eating goes much further than satisfying hunger; eating is a social factor that works as a means of relationship between people within a culture, with behaviors towards eating acquired in the family and social context^16^.

Among the non-modifiable risk factors, family history is a very useful variable to identify individuals at risk, since this disease occurs more frequently in the relatives of an individual diagnosed, than in those who are not. In addition, it is recognized that relatives of people with diabetes follow lifestyle patterns similar to those of the diagnosed patients^17^, what increases the risk. The family becomes a mechanism that can favor or disfavor health. Family is a fundamental element in the development of health and self-care behaviors, it represents emotional, affective, adaptive, informative, economic and functional support^18^. People at risk require knowledge, tools and skills for self-care, so that health professionals can encourage behaviors.

Tamaulipas is one of the states with the highest prevalence of type 2 diabetes mellitus in Mexico,^3^ and there is a knowledge gap regarding the pre-condition that prediabetes represents and the importance of the family context. The understanding of this phenomenon in the south of the state, through its risk factors, will help to provide an initial panorama of the problem, which will allow proposing strategies that favor, in this age group, the informed decisions and the resolution of problems at the family level, to achieve an impact in reducing the disease. Therefore, the present work was conducted with the following objective: to determine prevalence of obesity / overweight, physical activity, and prediabetes in adult children of people with diabetes mellitus type 2; identify differences according to sociodemographic variables (sex, age, occupation, and type of family) and describe the relationship of obesity / overweight and physical activity with blood glucose and glycosylated hemoglobin (A1c).

## Methods

This article is derived from the first year of a network project whose population consisted of adult children of people with DM2. The data of this work correspond to the population attended in a Health Center of the South of Tamaulipas, Mexico. A cross-sectional descriptive design with a convenience sample of 30 families was used, from which 53 participants derived.

The inclusion criteria were to be a child of a person with DM2, and be 18 years of age or older. Children who lived in another city, and pregnant women, were excluded. Data collection was carried out from April to August of 2013, and began with obtaining the corresponding authorizations for the Ethics and Research Committees of the Facultad de Enfermería de la Universidad Autónoma de Tamaulipas and the Sanitary Jurisdiction No II. With the support of the Directorate of the Health Center of interest, a module was installed for people with type 2 diabetes where they were invited to participate in a 30-minute interview. The objective was explained to the participant, highlighting the benefits of the study; those who agreed to participate in the interview were given a Family Data Card (which included data on the person with diabetes and family members, including age, conditions and type of family). If the children met the inclusion criteria, the person with diabetes was asked for his/her telephone number and address. 

Contact with the adult children was made through a home visit; the purpose of the investigation was explained to them; those who accepted were requested to provide verbal and written informed consent. In a subsequent visit, anthropometric measurements (height, weight in kilograms, and waist circumference in centimeters) and body composition (percentage of body fat) were carried out through a TANITA Body Composition Monitor (BC-554) previously calibrated to determine obesity / overweight. Height was measured with a portable stadiometer and waist circumference with a fiberglass tape measure, both from the SECA brand. All measurements were made according to the recommendations of the Ministry of Health of Mexico^19^.

To evaluate overweight and obesity by BMI, the World Health Organization (WHO) criteria were used ^20^. Central obesity was determined based on WC, with the criteria proposed by the International Diabetes Federation^1^. The risk of cardiovascular diseases was determined according to the WC, using the WHO cut-off points, and validated cut-off points were used for the classification of obesity according to the percentage of body fat.

To determine PA, the short version of the IPAQ Questionnaire was applied^21^, which measures three specific types of activity in the last seven days that are walking, moderate activity, and vigorous activity. The continuous indicator of PA is expressed in MET-minutes / week. The METs are a way to calculate the energy requirements, it is calculated by multiplying the MET corresponding to the type of activity, by the minutes of execution in a day or a week. To obtain the total PA continuously, a summation of the METs-week / minutes was made using the Ainsworth formula^22^: eight “minutes of vigorous activity” on days of vigorous activity; for moderate activity the formula is four “minutes of moderate activity” days of moderate activity; and for walking, 3.3 “walk minutes” walk days.

To classify PA by categories, the level of PA was calculated: high, for those individuals who had vigorous activity at least three days a week, with at least 1500 MET min/week, or seven days of any combination of PA reaching at least 3000 MET-minutes / week; moderate, for those who had three or more days of intense activity of at least 20 minutes per day, or five or more days of moderate activity, or walking of at least 30 minutes per day, or five or more days of any combination of PA reaching at least one PA total of 600 MET-minutes / week; and lastly, low level for those who did not meet the above criteria^21^.

Afterwards, the participants were given an appointment to go to the laboratory after a 12-hour fast for a blood glucose test and A1C. The criteria proposed by the American Diabetes Association were used as a reference framework to determine prediabetes^4^ , in which an amount of 70 - 100 mg / dl of fasting glucose is considered “normal”, 100 - 125 mg / dl is considered “impaired fasting glucose or prediabetes”, and 126 or more is considered “diabetes”.

The A1C test was performed to establish a reference frame of the average blood glucose that the relatives had during the twelve weeks before the measurement. The criteria proposed by the ADA were used^4^ to determine the average A1c, where a value of 4% equals approximately 50 - 80 mg/dl of blood glucose, 5% equals 80 - 115 mg/dl, 6% equals 115 - 150 mg/dl, 7% equals 150 - 180 mg/dl, 8% equals 180 - 210 mg/dl, and 9% is 210 - 245 mg/dl.

When the laboratory results were obtained, a third appointment was made with the participants to deliver them personally in their homes or in the Health Center; family education information on healthy eating and exercise was provided and they were thanked for their participation. Those who were detected with prediabetes, in addition to educational guidance, were channeled to the Health Center to join the exercise group and stay under control. Those who were suspected of having diabetes were given an educational orientation aimed at the characterization of the disease, carbohydrate counting, PA, and they were channeled with their family doctor to confirm the diagnosis and, if necessary, initiate the treatment of the disease and the relevant care. 

For the analysis of the data, the program used was the *Statistical Package for the Social Sciences* - SPSS, version 17.0. Descriptive statistics were used through measures of central tendency, dispersion, frequencies, and percentages, which allowed determining the prevalence of obesity, PA, and prediabetes. The normality of the data was verified with the Kolmogorov-Smirnov test. To identify differences in obesity / overweight and PA according to sex and age, the Mann Whitney *U*-test was used and for the differences according to occupation and family type, the Kruskal Wallis test was used. To determine the relationship of obesity (BMI, waist circumference, percentage of body fat) and PA (continuously) with blood glucose and A1C, non-parametric statistics were used, specifically the Spearman correlation test. 

This study adhered to the Regulation of the General Law of Health regarding research^23^.

## Results

The sample consisted of 30 families; the majority were nuclear families (Table 1).

**Table 1:** Type of family to which the adult children of people with diabetes belonged. Tampico, Tam., Mexico, 2013

	*f*	*%*
Nuclear	22	74
Single-parent	1	3
Aggregate	3	10
Mixed or reconstituted	4	13
Total	30	100

In total, there were 53 participant adult children of people with DM2. The sociodemographic characteristics is presented in Table 2, where the occupation of housewife and the informal sector, the youngest age group, and the female sex stand out.

**Table 2:** Sociodemographic characteristics of the adult children of people with diabetes according to sex, age, schooling, marital status, and occupation. Tampico, Tam., Mexico, 2013

Characteristic		Frequency (n= 53)	%
Sexo			
	Male	21	39.6
	Female	32	60.4
Age			
	From 18 - 30 years	30	56.6
	From 31 - 60 years old	23	43.4
Schooling			
	Elementary, incomplete	2	3.8
	Elementary	8	15.1
	Junior high school	19	35.8
	High school or equivalent	15	28.3
	College, incomplete	5	9.4
	College	4	7.5
Marital status			
	Single	18	34
	Married	20	37.7
	Separated	3	5.7
	Domestic partner	8	15.1
	Single mother	3	5.7
	Widow/Widower	1	1.9
Occupation			
	Public sector	1	1.9
	Employee in private company	18	34
	Worker in private company	6	11.3
	Established own business	1	1.9
	Informal sector	10	18.9
	Teacher	1	1.9
	Housewife	16	30.2

To determine the prevalence of obesity / overweight, PA, and prediabetes, the continuous descriptive data of their indicators are presented first. See table 3

**Table 3:** Descriptive data of obesity, physical activity and blood glucose of the adult children of people with diabetes. Tampico, Tam., Mexico, 2013.

Indicators		Male			Female		Total
		*Mean*	*SD*		*Mean*	*SD*	
Obesity							
	BMI*	27.39	4.28		26.90	5.59	27
	WC^†^	95.50	16.29		86.94	10.61	90
	Body fat	24.21	7.14		31.85	8.77	28.8
Physical activity							
	Total METs^‡^	3352	3091		1884	1726	2465
	Intense	373	846		442	1166	415
	Moderate	1428	1882		481	956	856
	Low	1550	1661		960	1230	1193
Prediabetes							
	Fasting glucose	91.74	15.65		100	31.84	96

*BMI: Body mass index; ^†^WC: Waist circumference; ‡METs: Continuous indicator of physical activity per week / minutes.

Regarding obesity / overweight, BMI was observed in the adult children of people with DM2 at a combined prevalence of 64 %. Based on WC, 73.6% had abdominal obesity and 68% had an increased or substantially increased risk for cardiovascular diseases. Based on the percentage of body fat, 49% of the participants presented with obesity / overweight. In relation to PA, a prevalence (according to METs-week / minutes) of 32.1% for low activity, 32.1% for moderate activity, and 35.8% for intense activity was observed. A total of 75% of the participants had a fasting blood glucose within normal, 19% had an altered glucose (for prediabetes), and 6% were strongly suspected of diabetes.

To determine differences in obesity and PA according to sex, age, occupation, and type of family, men had a significantly *(U = 219, p= 0.03)* greater WC than women. The women showed a significantly (*U = 142, p <0.01)* higher percentage of body fat than men. The oldest (between 18 - 30 years) had a higher body fat percentage (*U* = 195, *p* = 0.01). 

Based on occupation, significant differences X^2^ *(6) = 14.64, p=.02)* of obesity were found only in the body fat indicator, where those who were teachers (*Median* = 45.00), those who had established a business (*Mean*= 36.10), and housewives (*Mean*= 33.34) had a higher percentage. Regarding the type of family, the aggregate family showed a larger BMI, higher percentage of body fat, and greater WC (*p* <0.05), compared to the other family types (nuclear, single parent, and extended).

In PA, a difference was found *(U= 218, p=0.02)* only in moderate activity, where more activity was observed in men than in women. No significant differences in PA were found according to age or occupation (*p* > 0.05). 

Regarding the relationship between obesity / overweight with blood glucose and A1, the data of their indicators are presented in the Table 4.

**Table 4:** Correlation between anthropometric variables and body composition, physical activity with blood glucose and A1C * in adult children of people with diabetes. Tampico, Tam., Mexico, 2013

Variables	Blood glucose			A1C*	
	*r*	*p*		*r*	*p*
BMI^†^	0.158	0.289		0.417	0.004
WC^‡^	0.165	0.263		0.394	0.006
Body fat	0.336	0.022		0.494	0.000
Low PA^§^	-0.056	0.707		-0.189	0.199
Moderate PA	-0.016	0.914		-0.138	0.350
Intense PA	-.135	0.362		-0.285	0.049

*A1C: Glycosylated hemoglobin; †BMI: Body mass index; ‡Waist circumference; §Physical activity by category.

## Discussion

One of the outstanding findings of this study was the high prevalence of obesity / overweight due to BMI, and abdominal obesity due to WC in adult children of people with diabetes, which is consistent with the 2012 National Health and Nutrition Survey in Mexico, where reports state that seven out of ten adults over 20 are overweight or obese, according to BMI and seven out of ten suffer from abdominal obesity according to WC^3^. Even though BMI did not show significant differences, one was observed in WC, where men had more centimeters than women; while women showed higher percentage of body fat. This finding shows health risks because the central adiposity is associated with metabolic and cardiovascular alterations secondary to obesity, it is also more frequent to find insulin resistance - a condition that will favor the presence of prediabetes and diabetes - in adults when the fat is accumulated in the abdomen^13^.

Participants with greater age, an occupation, with less PA had more body fat percentage, and those belonging to aggregate families also had a higher WC. This finding highlights the need to consider the transcendence of the family group. First, the simple fact of having a family history with DM2 in the primary line doubles the risk of developing the disease. Secondly, obesity and diabetes have strong environmental components, such as food and PA, where the family plays a crucial role, since its members generally share the same environment, which influences their behaviors related to health^18^. The family as a support network can represent a potential for the reduction of disease problems, since it promotes greater social participation and community inter-relations^24^.

About a quarter of the participants in this study had impaired glucose for prediabetes, and suspected DM2 with the fasting glucose test. This result is similar to that reported in Mexico,^2^ where 20% had their glucose altered in fasting for prediabetes. This also coincides with findings reported by other authors,^10^ in a study conducted in the northern area of the city of Jalisco, with a sample of 423 participants. The prevalence reported by these authors was around 20%. Our finding is also congruent with some international studies^9^. However, it is also recognized that the prevalence found in our study does not coincide with other international studies, such as those in the United States^5^ and England^6^, where prevalence of prediabetes was reported above 30%. A possible explanation of the differences could be the sample size. 

Blood glucose was related only to body fat, but not BMI, WC or PA; however, positive relationship of A1C with BMI, WC, body fat, and an inverse relationship was observed with intense PA. Considering that, the blood glucose and A1C figures are related, since the first reflects the glucose level that the person has now of the blood draw, and the A1C is a measurement that shows the mean of the glycemia over the last 10 - 12 weeks. This finding is relevant because it suggests that the higher the BMI, WC and body fat, the higher the average glucose in the previous weeks, and the higher the risk of developing glucose alterations. On the other hand, the inverse relationship between the A1C and intense PA is consistent with the literature that indicates that physical activity has physiological benefits, among which the control of blood glucose for the prevention of diabetes stands out^11^. In this study, it was observed that two of three (64.2%) participants registered between low and moderate PA, according to METs-week / minutes, and men had higher moderate PA than women, a situation that represents a risk factor for further alterations in glucose.

## Conclusions

Although the findings cannot be generalized due to the size of the sample, which is recognized as a limitation of this study, the diagnosis of prediabetes and diabetes in the apparently healthy population is a favorable condition for secondary prevention, as long as it addresses the risk factors present. This study allowed us to identify a high prevalence of obesity and overweight, as well as low PA, in adult children of people with diabetes.

Obesity and overweight are prediabetes condition, whose etiology have a double association with the family organization: the genetic inheritance imposes a dominant element on the transmission of DM2, and eating habits and physical activities that are modulated within the family group. Generally, people living with diabetes-prediabetes, overweight / obesity- receive interventions on the individual level, neglecting the family dimension, which is where the individual cases that make up the community are born. 

Health professionals need to perceive the phenomenon from a broad perspective that involves the social determinants of health; it is then necessary to adopt survival strategies within family groups, such as the modification of eating habits and physical activity through specific intervention programs. It is important that this phenomenon continue to be studied in future research and with representative samples where health professionals can intervene at the family level.
